# Abdominal Wall Hernias Following High-intensity Focused Ultrasound Therapy: Three Case Reports

**DOI:** 10.1055/a-2268-6986

**Published:** 2024-07-18

**Authors:** Woo Yeon Han, Yeongsong Kim, Pyeong Hwa Kim, Eun Key Kim

**Affiliations:** 1 Department of Plastic Surgery, Yongin Severance Hospital, Yonsei University College of Medicine, Yongin, Korea; 2 Department of Plastic Surgery, Asan Medical Center, University of Ulsan College of Medicine, Seoul, Korea; 3 Department of Radiology, Asan Medical Center, University of Ulsan College of Medicine, Seoul, Korea

**Keywords:** hernia, HIFU, uterine adenomyosis

## Abstract

Although many studies reported the safety and efficacy of high-intensity focused ultrasound (HIFU) therapy, there are still worries about internal organ injury. However, reports of abdominal wall hernias after HIFU therapy are rare.

We present three cases of abdominal wall hernias without skin injury after HIFU therapy in uterine adenomyosis or fibroids. The diagnosis was often delayed because of vague symptoms, inadequate clinical suspicion, and delayed proper image studies.

Abdominal wall hernia should be recognized as a possible complication after HIFU and be suspected when the patient presents with unordinary abdominal swelling and/or pain that lasts for more than a few months after the procedure.

## Introduction

Uterine adenomyosis is a common gynecologic disorder affecting females of reproductive age. Increasing focus on uterine preservation and advances in technology have led to the development of minimally invasive treatment options. High-intensity focused ultrasound (HIFU) therapy uses focused ultrasonic energy for coagulative necrosis of the target area without affecting adjacent tissue. Since ultrasound-guided HIFU therapy was approved by the U.S. Food and Drug Administration in 2004, it has been widely used for clinical treatment of uterine adenomyosis. 1 Although many studies reported the safety and efficacy of HIFU therapy, there are still worries about internal organ injury. However, reports of abdominal wall hernias after HIFU therapy are rare. 1 2 3 4 We present three cases of abdominal wall hernias without skin injury after HIFU therapy in uterine adenomyosis or fibroids. This study was approved by the Institutional Review Board of the authors' institution (2202-0895). Informed consent has been obtained from the patient for the use of clinical photographs and medical images.

## Cases

### Case 1

**Fig. 1:**
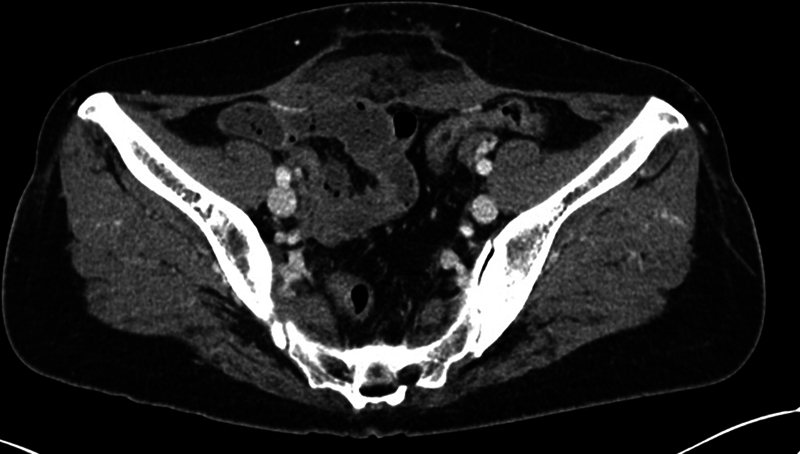
Axial contrast-enhanced CT image of case 1 showing abdominal wall hernia without overlying skin injury after HIFU therapy. Rectus abdominis muscle injury, fascia rupture, and subcutaneous fluid collection were noted 1 year after HIFU. CT, computerized tomography; HIFU, high-intensity focused ultrasound.

A 51-year-old female patient visited the outpatient clinic with painful abdominal swelling lasting for 1 year. She was asymptomatic except for abdominal swelling. She was a parous woman without diabetes (BMI = 23.5 kg/m ^2^ ). She had previously received HIFU therapy for the management of uterine adenomyosis 15 months ago. Abdominal pain and swelling had not subsided for a few weeks after the procedure, and magnetic resonance imaging was recommended for screening for possible complications of HIFU. The clinician found rectus abdominis muscle injury with fascial rupture and subcutaneous fluid collection but intact skin barrier. A follow-up computerized tomography (CT) after 1 year showed no improvement in musculofascial injury and aggravated overlying subcutaneous fat necrosis and localized fluid collection ( Fig. 1 ). Patient is reluctant to undergo open surgery. Regular follow-up of her worsening symptoms is planned.

### Case 2

**Fig. 2:**
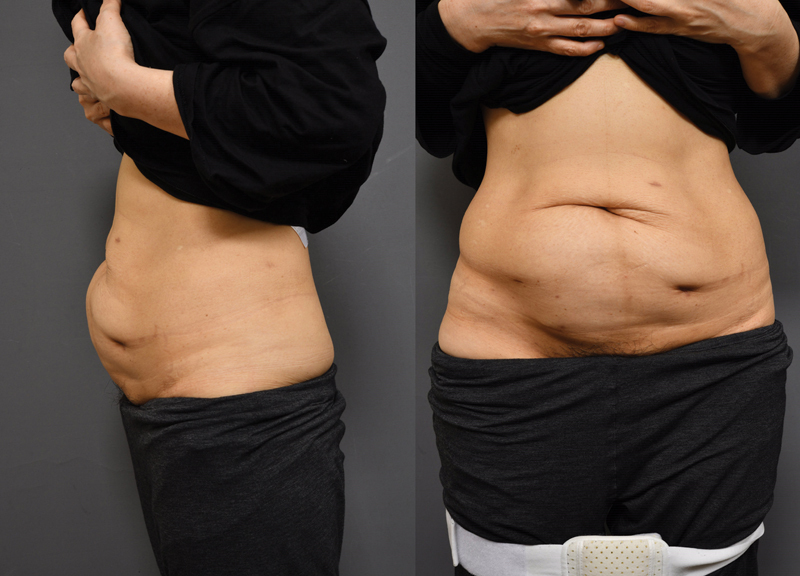
Abdominal wall bulging 12 months after HIFU therapy (lateral view, anteroposterior view). HIFU, high-intensity focused ultrasound.

**Fig. 3:**
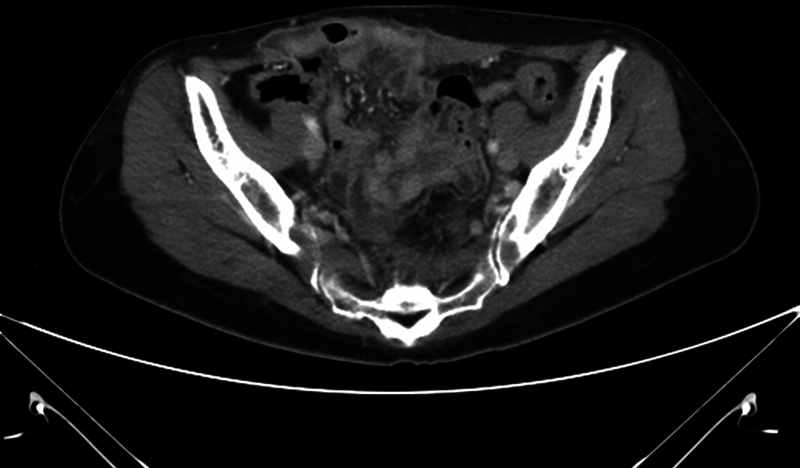
Axial contrast-enhanced CT image of case 2 showing abdominal wall hernia without overlying skin injury after HIFU therapy. Abdominal wall hernia with rectus abdominis muscle atrophy was noted. CT, computerized tomography; HIFU, high-intensity focused ultrasound.

**Fig. 4:**
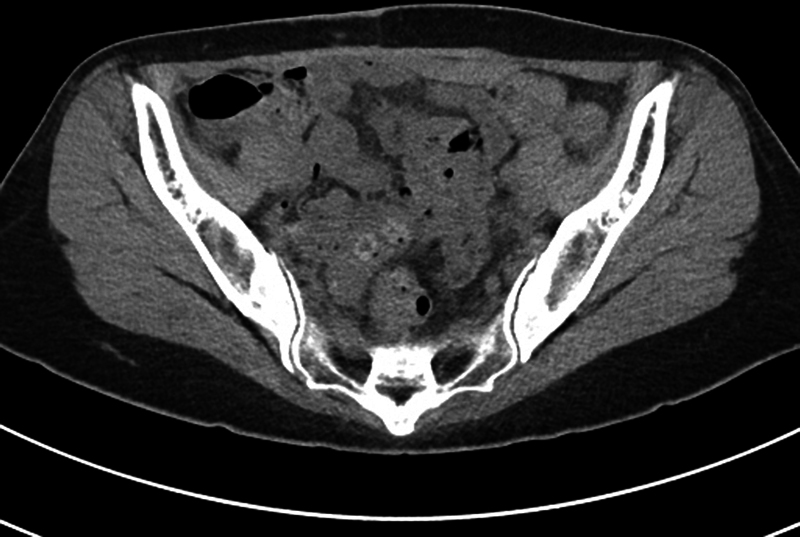
Axial contrast-enhanced CT image of case 2. Postoperative 3 months. CT, computerized tomography.

A 54-year-old female patient visited the outpatient clinic for treatment of an abdominal wall hernia ( Fig. 2 ). She was a parous woman without diabetes (BMI = 20.6 kg/m ^2^ ). She had previously received HIFU therapy for debulking a large-sized uterine fibroid 12 months ago. The patient first returned to ordinary life without any obvious complications after HIFU. Four months later, she felt like something was breaking in her abdomen when lifting heavy objects. She found lower abdominal bulging and the gynecologist suspected recurrent myoma and recommended CT imaging. On the CT scan, 10-cm-sized abdominal wall hernia and rectus abdominis muscle atrophy containing small bowel loops through the right anterior aspect of the abdominal wall was found ( Fig. 3 ). Patient underwent abdominal wall reconstruction with abdominal flap elevation, musculofascial coaptation followed by acellular dermal matrix onlay graft. Deep fascia disruption with fluid collection was noted during operation. No complications or recurrence at 10 months postoperatively ( Fig. 4 ).

### Case 3

**Fig. 5:**
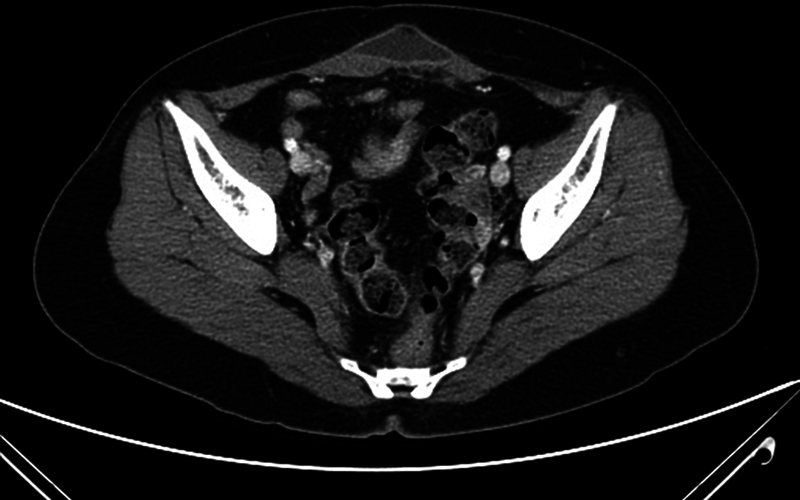
Axial contrast-enhanced CT image of case 3 showing abdominal wall hernia without overlying skin injury after HIFU therapy. Rectus abdominis muscle injury and the overlying fluid collection lasted for more than 1 year in spite of repeated needle aspiration. CT, computerized tomography; HIFU, high-intensity focused ultrasound.

**Fig. 6:**
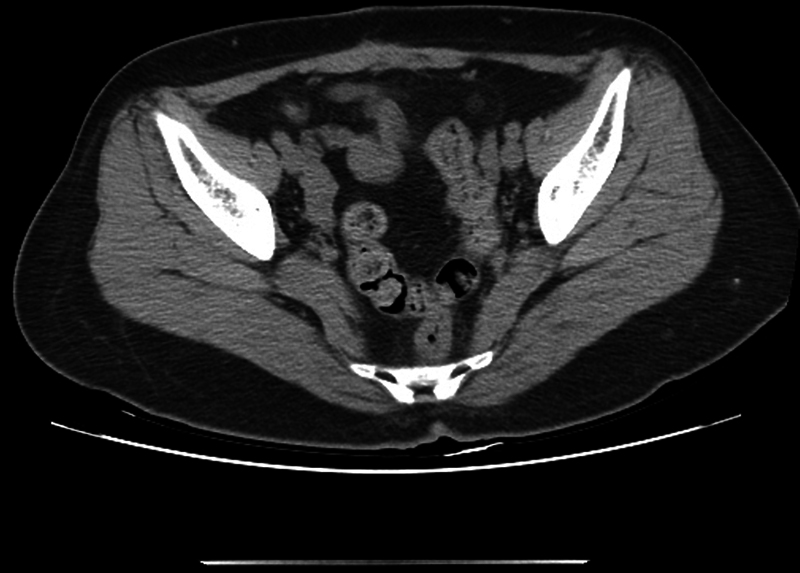
Axial contrast-enhanced CT image of case 3. Postoperative 12 months. CT, computerized tomography.

A 46-year-old female visited the outpatient clinic for fluctuation in her abdomen. She was asymptomatic, except for abdominal fluctuation with normal C-reactive protein level. She was a parous woman without diabetes (BMI = 24 kg/m ^2^ ). She underwent HIFU therapy for uterine adenomyosis 2 years ago. Minor abdominal bulging developed soon after HIFU therapy, and slowly progressed thereafter. One year later, she was suffering from recurrent cellulitis on her lower abdomen, and a CT scan found subcutaneous fluid collection with 9-cm-sized rectus abdominis muscle injury. Needle aspiration and antibiotics therapy was repeatedly prescribed for symptomatic relief without any definite resolution of the situation ( Fig. 5 ). Patient underwent abdominal wall reconstruction with abdominal flap elevation, musculofascial coaptation followed by acellular dermal matrix onlay graft; 9 × 6 cm ^2^ -sized hernia sac and fluid collection was found during operation. No complications or recurrence at 1 year postoperatively ( Fig. 6 ).

Period of symptom development after HIFU, hernia diagnosis after HIFU ( Table 1 ) show the relative delay in the diagnosis of abdominal wall hernia after HIFU therapy in cases 1 to 3.

**Table 1:** Period of symptom development after high-intensity focused ultrasound, hernia diagnosis after high-intensity focused ultrasound

	Symptom development after HIFU	Hernia diagnosis after HIFU
**Patient 1**	A few weeks	3 months
**Patient 2**	4 months	7 months
**Patient 3**	A few days	2 years

Abbreviation: HIFU, high-intensity focused ultrasound.

## Discussion

HIFU is a noninvasive therapy that uses nonionizing ultrasonic waves to heat or ablate tissue. HIFU delivers higher time intensities of ultrasound in focal regions than diagnostic ultrasound. It generates heat of over 60°C in tissues, causing coagulation necrosis and cavitation, which is the creation of a gas cavity causing disruption to cell membranes. 5 Because of its advantages, it is used in many medical fields, including the treatment of uterine adenomyosis and fibroids. 6 7 Several studies have reported the safety and efficacy of HIFU therapy. 1 2 3 4 Liu et al reported that ultrasound-guided HIFU significantly improves the quality of life safely and with lower cost than hysterectomy. 8 For those reasons, HIFU therapy is now a preferred treatment option of uterine adenomyosis and fibroids.

However, previous studies have also reported complications of HIFU therapy. Chen et al reported that out of 9,998 patients, lower abdominal pain occurred in 225 patients (2.25%) within 7 days and was graded as Class A according to the Society of Interventional Radiology classification system. However, an abdominal wall hernia occurred in only one patient (0.01%) after 90 days and was graded as Class D, needing major therapy with prolonged hospitalization. 2 However, it was uncertain whether proper physical examinations and/or imaging studies were performed in all patients for evaluation of abdominal wall hernias.

During HIFU procedure, reflection of high-energy by intestinal gas or skeletal structure can cause tissue damage to adjacent tissue near target organ. However, unlike other complications, patients with abdominal wall hernias after HIFU can have deep tissue damage including abdominal muscles without realizing it, as they experience only vague symptoms such as abdominal pain or bulging but no visible skin injury. In current cases, we found that abdominal wall hernias can occur after HIFU without recognition of the abdominal wall weakening. Even worse, the patients who receive HIFU for uterine adenomyosis often have diastasis recti and subsequent abdominal bulging already, and they are used to abdominal discomfort and pain due to their previous uterine adenomyosis, all of which further delay the diagnosis of abdominal wall hernia. There is no consensus on the risk factors for abdominal wall complications following HIFU.

To the best of our knowledge, this is the first case report of abdominal wall hernia without skin injury after HIFU therapy. The diagnosis was often delayed because of vague symptoms, inadequate clinical suspicion, and delayed proper image studies. Abdominal wall hernia should be recognized as a possible complication after HIFU and be suspected when the patient presents with unordinary abdominal swelling and/or pain that last for more than a few months after the procedure.
